# Supplementary material for: Patterns of Intron Gain and Loss in Fungi
Source: PLoS Biol. 2004 Nov 30;2(12):e422. doi: 10.1371/journal.pbio.0020422 (PMC532390; doi:10.1371/journal.pbio.0020422)
Supplement: Table S1 — Also available at http://genes.mit.edu/NielsenEtAl/. (4.3 MB ZIP). [file pbio.0020422.st001.zip › NielsenEtAl/html/764.html]

AN2759.1.NCU02428.1.MG03587.1.FG09853.1


```
 CLUSTAL W (1.82) Multiple Sequence Alignments - Introns Inserted


Sequence 1: NCU02428.1	257 aa
Sequence 2: MG03587.1	252 aa
Sequence 3: FG09853.1	250 aa
Sequence 4: AN2759.1	260 aa
Alignment Length: 264 aa
Number Identitical Residues: 183 aa
Alignment Score (without introns) 7695


MG03587.1 	------------MAG~TQSLPPPELPQLVAEQHQPIPSSDKDSRRLIVVLSNASLETYKA
NCU02428.1	-----MSNHDVTMAG~TQSLPPPALPQLVAEQHMPIPPTDKDTKRLIVVLSNASLETYKA
FG09853.1 	-----MS----ETLG~TQSLPPPSLPQLVAEQSTPIPPTDKDSQRLIVVLSNASLETYKA
AN2759.1  	MSESVQVAGKRKRAR1TQSCPPPELPQLVAEQHVPIAPNDKETQRLIVVLSHASLETFKA
          	 :.:     .      *** *** ********  **...**:::*******:*****:**

MG03587.1 	SHSAGPNRMGAAPREEKYSLLNSDEHIGVMRKMNRDISDARPDITHQ0CLLTLLDSPINK
NCU02428.1	SH-GGSGRMGMQ-REEKYSLLNSDEHIGVMRKMNRDISDARPDITHQ0CLLTLLDSPVNK
FG09853.1 	SH-GGTSRN----REDKYSLLNSDEHIGVMRKMNRDISDARPDITHQ0CLLTLLDSPINK
AN2759.1  	SHGGRNGTA----RDEKYSLLNSDEHIGVMRKMNRDISEARPDITHQ0CLLTLLDSPVNK
          	**..  .      *::**********************:******** *********:**

MG03587.1 	AGKLQIYIQTAKGVLIEVSPSVRIPRTFKRFAGLMVQLLHRLSIRSTNSQEKLLRVIQNP
NCU02428.1	AGRLQIYIQTAKGVLIEVSPAVRIPRTFKRFAGLMVQLLHRLSIKGTNSQEKLLKVIQNP
FG09853.1 	AGKLQIYIHTAKGVLIEVSPSVRIPRTFKRFAGLMVQLLHRLSIRSTNSNEKLLRVIQNP
AN2759.1  	AGKLQIYIHTAKGVLIEVNPSVRIPRTFKRFAGLMVQLLHRLSIRSTNSQEKLLKVIKNP
          	**:*****:*********.*:***********************:.***:****:**:**

MG03587.1 	ITDHLPPHCRKVTLSFDAPVVRVRDYIDSLDSKESICVFVGAMAKGADNFADEYVDEKIG
NCU02428.1	ITDHLPPNCRKITLSFDAPVVRVREYIDALGENESICVFVGAMAKGADNFADEYVDEKIS
FG09853.1 	ITDHLPPNCRKVTLSFDAPLVKVREYVESVNSKDSICVFVGAMAKGEDNFADSLVDEKIS
AN2759.1  	ITDHLPPNCRKVTLSYEAPVVRVKDYIESLGPKESICIFVGAMAKGQDDFADSFKDDTIS
          	*******:***:***::**:*:*::*::::. ::***:******** *:***.  *:.*.

MG03587.1 	ISHYSLSASVACSKFCHAAEDCWDIL
NCU02428.1	ISNYSLSASVACSKFCHAAEDAWDII
FG09853.1 	ISNFSLSASVACSKFCHAAEDVWDIM
AN2759.1  	ISNYSLSASVACSKFCHAAEEVWDIL
          	**::****************: ***:
```
